# The prevalence and risk factors for physical impairments in Chinese post-cancer treated breast cancer survivors: a 4 years’ cross-sectional study at a single center

**DOI:** 10.1038/s41598-023-45731-x

**Published:** 2023-10-27

**Authors:** Dan Chen, Li Li, Liu-Ya Jiang, Jie Jia

**Affiliations:** Department of Rehabilitation Medicine, Shanghai Jing’an District Central Hospital, Shanghai, China

**Keywords:** Cancer, Health care, Risk factors, Signs and symptoms

## Abstract

The incidence of breast cancer in China was 19.2% in 2018, with a five-year survival rate of up to 80%. The impairments that may result from breast cancer treatment, such as lymphedema, pain, and symptoms related to nerve damage, could have long-term side effects. Its prevalence and symptom profile have been commonly reported in various countries, but such data are rarely available for China. Physical function was assessed in 138 breast cancer survivors (BCSs) in the study. The prevalence of lymphedema (65.9%) was higher than that of pain (31.2%), shoulder range of motion (ROM) restriction (20.3%), grip strength restriction (GSR) (21.7%) and paresthesia (11.6%). These impairments mainly appeared within 28 months after breast cancer diagnosis, but could happen in 10 years. Carcinoma in situ and radiotherapy (RT) were related to the occurrence of lymphedema (respectively B = -1.8,* p* = 0.003; B = 1.3, *p* = 0.001). RT and delayed rehabilitation time (DRT) may increase the severity of lymphedema (respectively *p* = 0.003, *p* = 0.010). Breast conserving surgery (B = -2.1, p = 0.002) and the occurrence of AWS (B = 3.1, *p* = 0.006) were related to the occurrence of pain. The occurrence of brachial plexus injury (BPI) (B = 3.1,* p* < 0.001) and pain (B = 1.9, *p* = 0.002) improved the occurrence of shoulder ROM restriction. The occurrence of BPI (B = 3.6, *p* < 0.001) improved the occurrence of GSR. The occurrence of pain (B = 2.1, *p* = 0.001) improved the occurrence of paresthesia. These findings prompt us to further investigate the actual rehabilitation needs of survivors and the specific barriers to rehabilitation in the following research.

## Introduction

According to global cancer statistics 2020^1^, the incidence of breast cancer has surpassed lung cancer to become the most common malignant tumor worldwide. The incidence of breast cancer in Chinese women was 19.2% and showed an upward trend in the 2018 survey^2^. Moreover, the population of China has exceeded 1.4 billion, and the incidence of breast cancer shows a younger trend in the affected population^3^. Therefore, during clinical follow-up, which is the early detection of recurrence or metastasis, a higher attention should be paid to the function, quality of life (Qol) and return to work (RTW) of breast cancer survivors (BCSs)^4^. Initially, breast cancer patients were treated with the classic radical mastectomy^5^, which caused frequent arm problems, such as lymphedema, reduced range of motion (ROM) of the shoulder, pain, numbness and muscle weakness^6^. Surgery evolved into modified radical mastectomy^7,8^ causing less harm to the patients.

Mastectomy may also cause shoulder impingement and rotator cuff disease^4,9–14^, which can further lead to shoulder pain^15^, reduced range of motion, and muscle strength^16^. Sentinel lymph node biopsy (SLNB) and axillary lymph node dissection (ALND) not only increase the risk of arm damage, lymphedema, and chronic pain syndrome^17^, but damage the intercostal brachial nerve and other brachial plexus nerves, causing abnormal sensation^18^. Abnormal sensations may occur in the chest wall, armpit, upper arm, and sometimes in the upper back and around the affected breast^19,20^. Radiation may also aggravate this injury^18^. In addition, adjuvant treatments, such as radiation, can also cause fibrosis and negatively affect the microvascular system^21^, while the dose or volume of radiation can affect the outcome of shoulder function, including pain, stiffness^22^, and mobility.

The incidence and presentation of above-mentioned sequelae vary somewhat due to assessment criteria and study design. In a 2018 cross-sectional study, Hamood et al. reported that 84% of BCSs who were members of the “Leumit” Medical Fund in Israel had chronic pain and 63% had paresthesia^19^. In a prospective observational study in the United States, 10% BCSs had decreased ROM, 49% had pain, and 47.1% had numbness at 12 + months after surgery^23^. A 10-year cohort study in the United States reported that chronic lymphedema occurred in 5/108 (4.6%) and 40/115 (34.8%) of the SLNB and ALND groups, respectively^24^. Monleon et al. found that compared with SLNB surgery, the strength of internal rotator muscle after ALND in breast cancer decreased significantly in the first month, and still did not recover after 1 year of follow-up, with an average difference of 2.26 kg^25^. A one-year prospective observational study in Korea found that shoulder strength and ROM returned to baseline one year after latissimus dorsi flap surgery^26^. In a 5-year longitudinal study, Boquiren et al., Canada, found that restricted ROM and pain, except for lymphedema, peaked one year after surgery and then declined significantly after 5 years^27^. However, there are few studies on the occurrence and development of postoperative sequelae of breast cancer in China, which is not conducive to our understanding of the actual long-term rehabilitation needs of BCSs, so it is unable to provide an effective referral system and timely rehabilitation^28^.

The main objective of this study was that we attempted to determine the incidence and prevalence of Arm morbidity (AM) including lymphedema, reduced ROM of the shoulder, pain, muscle weakness and paresthesia among BCSs in China. The secondary objective was to assess the possible risk factors associated with the development of upper limb disease and to analyze the potential factors affecting the recovery of upper limb function.

## Methods

### Study design and participants

A cross-sectional study was conducted and all measurements took place in the department of rehabilitation medicine, Jing'an District Central Hospital, Shanghai. The rehabilitation evaluation was conducted for breast cancer patients who came to the hospital for the first time from January 1, 2017 to December 31, 2020, and their physical function was obtained and their rehabilitation needs were collected. Patients who could not participate normally due to cognitive impairment, had a history of upper limb neuromuscular disease or congenital lymphedema before breast cancer diagnosis, were diagnosed with bilateral breast cancer, and did not agree to participate in this study were excluded. This study was reviewed and approved by Guojun Zhu, Hong Wang and Min Gong etc. who are the members of Medical Ethics Committee of Shanghai Jing'an District Central Hospital. The ethical approval number is 2019–2023. Patients were informed of the contents of this study and their freedom to participate in the study, and they were not treated differently even if they did not participate in the study. Participants could withdraw from the study at any time. All participants in this study signed an informed consent form, and they were assessed by researchers who had been trained in the assessment criteria by professors from the University of Sydney.

### Procedures and measures

Participants took a subjective questionnaire for demographic information, clinical characteristics mainly including axillary web syndrome (AWS) and brachial plexus injury (BPI), pain, paresthesia, allodynia, and phantom sensations, and were measured for AM including lymphedema, pain, ROM, and muscle strength by trained clinical research assistants. The training included attending a two-day workshop led by a certified rehabilitation therapist in China. The workshop included a comprehensive, illustrated assessment manual, field operations, and real-time feedback from trainers.

### Demographic and clinical characteristics

Demographic information included sex, age, height and weight (used to calculate body mass index (BMI)). Clinical information included cancer type, surgery type (mastectomy, modified radical mastectomy, breast conserving surgery), lymph node dissection type (axillary, sentinel), numbers of positive nodes removed, treatment type (radiation, chemotherapy, hormonal therapy). Based on the medical model that cancer rehabilitation should be intervened after cancer diagnosis, we defined the delayed rehabilitation time as the time between the first rehabilitation evaluation and cancer diagnosis. In order to explore the time distribution of dysfunction, we calculated the time of patient's first dysfunction based on the time of diagnosis.

### Measurement of grip strength and ROM

Hand-grip strength test was performed as an indicator of overall strength. It was measured using J-Tech grip strength device (JTech Medical, Midvale, Utah). The tracker computerized grip dynamometer is a wireless grip device that provides a reliable grip force assessment. The units of it were displayed in kilograms of force. In previous studies, test–retest reliability of J-Tech equipment has been examined, and it was excellent at high ICC value, which ranged from 0.954 to 0.973^29^. And research assistant gave a description of the hand grip strength test procedure and performed 3 trials on the patients and calculated the mean of the 3 trials. Participants were given a 15-s break before the next test. During the test, the grip force was pressed for 2–3 s to ensure maximum grip strength. Measurements were taken in the standard position of the elbow at 90%, the forearm neutral, and the wrist in neutral deviation, and both hands were tested as described above. Grip strength restriction (GSR) was defined as the difference in the force between the affected and unaffected sides.

A goniometer was used to measure shoulder flexion, extension, abduction, horizontal position-internal rotation and external rotation in an upright or sitting positon. The movement was measured to a sensation of pain or a degree that the patient's arm could no longer move. Full shoulder abduction starts with arms alongside your sides, palms facing forward and fingers pointing at your toes. In the coronal semicircle motion, move the arm until the hand is up and the fingers point to the ceiling. Complete shoulder flexion starts in the same position as abduction. In a sagittal semicircle motion, arms move until the hand is up and the fingers point to the ceiling. Shoulder extension begins with shoulder flexion, and in a sagittal semicircle motion, the arm moves backward to the maximum angle. The starting position of horizontal position-internal rotation and external rotation is 90° abduction of the shoulder joint with elbow flexion parallel to the ground. Internal rotation occurs by moving the fingers down until they point to the floor, and external rotation occurs by moving the fingers up until they point to the ceiling. ROMs were measured on both the affected and non-affected sides. ROM restriction was defined as the difference in the degree of rotation between the affected and unaffected sides. The possible fractional range for both flexion and abduction are 0° to 180°. The possible fractional range for extension is 0°–60°. The possible fractional range for both horizontal position-internal rotation and external rotation 0°–90°.

### Measurement of lymphedema

Arm measurements were undertaken using a retractable Jobst non-stretched soft tape measure commencing at the midpoint of the ulnar styloid set as the 0 cm mark and then at 10 cm intervals up to 40 cm proximal to the ulnar styloid. To improve diagnostic sensitivity, we consider lymphedema to be present when the circumference difference measured at a single point is greater than 1 cm.

### Measurement of pain

The Numerical Rating Scales (NRSs) was used to measure pain, which was a simple and common way to assess changes in pain intensity. With the NRSs, participants were asked to rate their average pain intensity over the last 7 days by selecting a single number from 0 to 10. The repeatability of the Visual Analogue Scale (VAS) is good as can be seen by correlation coefficients ranging from 0.97 to 0.99^30^. The correlation between the NRSs and VAS was statistically high (r = 0.93)^31^.

### Analyses

We described participants' demographic information, history of cancer treatment, incidence of sequelae, time of impairments occurrence and rehabilitation delay time. At the same time, the degree and extent of lymphedema, pain, limited ROM and muscle strength, and paresthesia were described. Among them, frequencies and percentages were used for categorical variables, continuous variables used means and standard deviations, or medians and quartiles when the data was not normally distributed. Chi-square tests were used to analyze the factors influencing the occurrence of lymphedema, then a possible model was obtained by multivariate logistic regression analysis. Kruskal–Wallis Tests were used to analyze the factors influencing the severity of lymphedema, then a possible model was obtained by multivariate linear regression analysis. Multivariate logistic regression was used to analyze the factors influencing the occurrence of pain, shoulder mobility limitation, grip strength limitation and paresthesia. Statistical significance was determined with *p* values of < 0.05, and all analyses were performed using SPSS version 25.0 (IBM SPSS Statistics, Armonk, NY, USA).

## Results

### Characteristics of participants

We recruited 169 patients for participation in this study from January 1, 2015, to December 31, 2021. Thirty-one of these patients did not meet the inclusion criteria. Of these, twenty-two of patients had rotator cuff injury prior to diagnosis, and nine had bilateral breast cancer. Therefore, the final population in the intent to analysis included 138 patients. The characteristics of the 138 remaining participants in the study were listed in Table 1. Most of the participants were aged 51–70, accounting for 65.9%. According to the Chinese BMI standard, 46.4% of the participants were in a normal criterion, 36.2% were overweight and 14.5% were obese. Infiltrating ductal were found in 84.1% of BCSs who seek rehabilitation. Among the patients, 31.2% underwent mastectomy, 54.3% underwent modified radical mastectomy, and 14.5% underwent breast-conserving surgery. ALND was performed in 89.2% of patients. The numbers of lymph nodes dissected in 45.6% patients more than 15. There were 89.9% of patients received chemotherapy, 56.6% received radiotherapy and 62.3% received endocrine therapy. The sequelae were lymphedema (65.9%), pain (31.2%), shoulder ROM restriction (20.3%), GSR (21.7%) and paresthesia (11.6%). The participants had a 6.5% incidence of AWS and 10.9% incidence of BPI. Possible sequelae occurred within 28 months of breast cancer diagnosis in 74.6% of participants. After the occurrence of sequelae, 80.4% of the participants would try to seek relevant treatment within 0–20 months, while 19.6% of the participants did not find a way to face the unavoidable sequelae until 70 + months (Table 1).

**Table 1:** Characteristics of participants.

Characteristics	*N*	%
Sex		
Female	137	99.3
Male	1	0.7
Age at inclusion		
32–40	15	10.9
41–50	21	15.2
51–60	42	30.4
61–70	49	35.5
71–85	11	8.0
BMI		
< 18.5	4	2.9
18.5–23.9	64	46.4
24–27.9	50	36.2
≥ 28	20	14.5
Cancer type		
Infiltrating ductal	116	84.1
Carcinoma in situ	15	10.9
Others	7	5.0
Surgery type		
Mastectomy	43	31.2
Modified radical mastectomy	75	54.3
Breast conserving surgery	20	14.5
Lymph node procedure		
ALND only	95	68.8
SLND only	9	6.5
ALND and SLND	28	20.4
Does not apply	6	4.3
Numbers of dissected lymph nodes		
0–1	35	25.4
2–14	40	29.0
15–18	34	24.6
19 +	29	21.0
Numbers of lymph node metastases		
0	86	62.3
1–2	19	13.8
3 +	33	23.9
Chemotherapy		
Yes	124	89.9
No	14	10.1
Radiotherapy		
Yes	80	58.0
No	58	42.0
Endocrine therapy		
Yes	88	63.8
No	50	36.2
Lymphedema		
Yes	91	65.9
No	47	34.1
Pain		
Yes	43	31.2
No	95	68.8
Shoulder ROM restriction		
Yes	28	20.3
No	110	79.7
Grip strength restriction		
Yes	30	21.7
No	108	78.3
Paresthesia		
Yes	19	13.8
No	119	86.2
Axillary web syndrome		
Yes	9	6.5
No	129	93.5
Brachial plexus injury		
Yes	15	10.9
No	123	89.1
Time of symptom occurrence after diagnosis, month		
0–28	103	74.6
29–70	14	10.1
71 +	21	15.3
Delayed rehabilitation time, month		
0–20	111	80.4
21–69	13	9.4
70 +	14	10.2

The time of delayed rehabilitation is from the onset of symptoms reported by the patient to the onset of rehabilitation.

### The cumulative prevalence of dysfunction and the time to first occurrence after diagnosis

**Figure 1:**
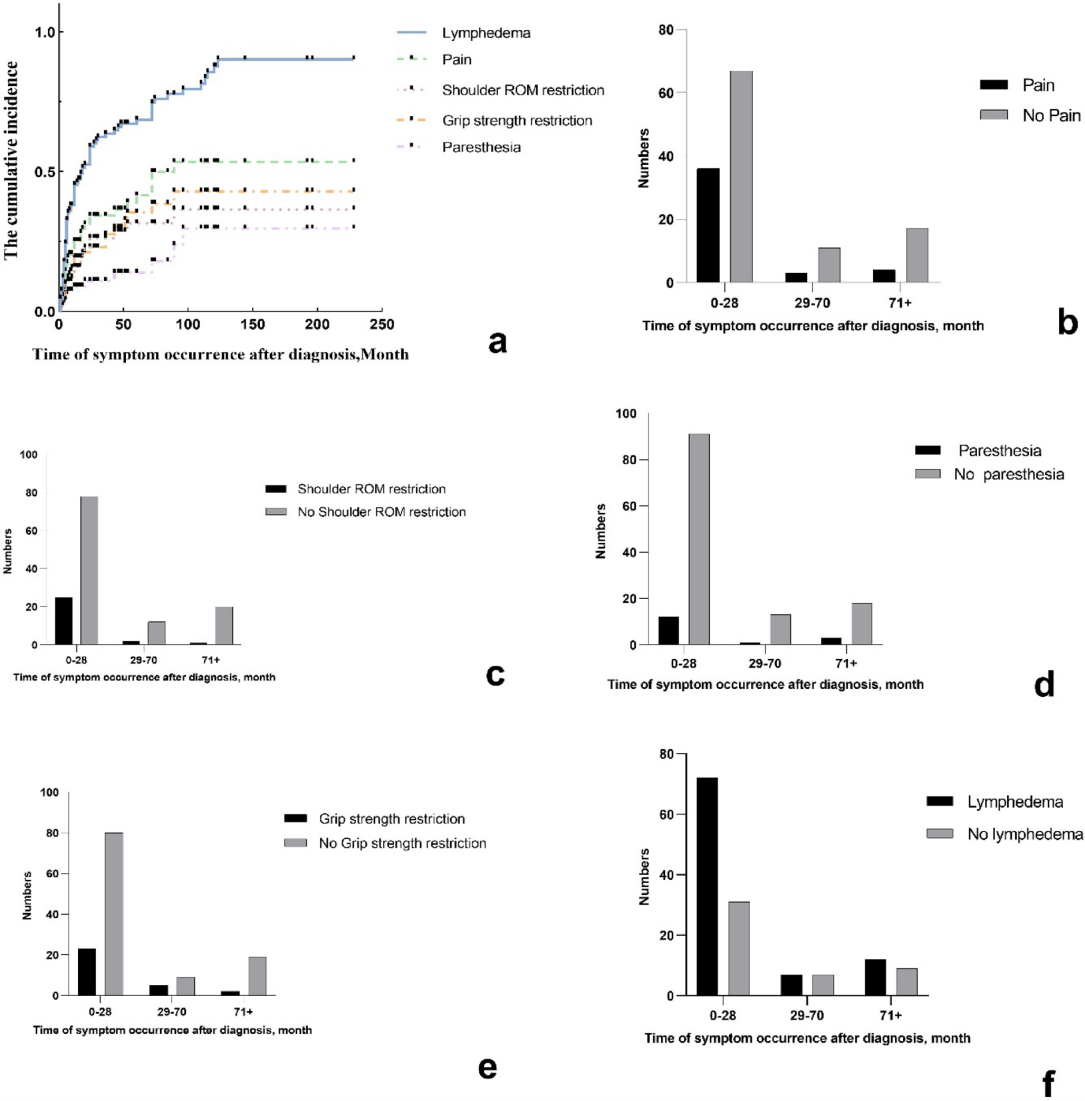
The prevalence of dysfunction and the time of first occurrence after diagnosis in breast cancer patients.

Figure 1a presented the cumulative prevalence of lymphedema, pain, shoulder ROM restriction, decreased grip strength and paresthesia. Although there was no statistically significant difference (*p* = 0.28), the incidence of pain in 0–28 months after treatment (35.0%) was higher than that in 29–70 months (21.4%) and 71 + months (19.0%) (Fig. 1b). The incidence of shoulder ROM restriction in 0–28 months after treatment (24.3%) was higher than that in 29–70 months (14.3%) and 71 + months (4.8%) (*p* = 0.12) (Fig. 1c). The incidence of paresthesia at 0–28 months after treatment (11.7%) was higher than that at 28–70 months (7.1%) and lower than that at 71 + months (14.3%) (*p* = 0.82) (Fig. 1d). The incidence of decreased grip strength in 0–28 months after treatment (22.3%) was higher than that in 71 + months (9.5%) and lower than that in 29–70 months (35.7%) (*p* = 0.18) (Fig. 1e). The incidence of lymphedema in 0–28 months after treatment (69.9%) was higher than that in 29–70 months (50.0%) and 71 + months (57.1%) (*p* = 0.21) (Fig. 1f).

### The degree and extent of lymphedema, pain, limited range of motion and muscle strength, and paresthesia

The arm circumference of participants at 10cm, 20cm, 30cm and 40cm above the wrist was more than 1cm, respectively was [1.90 (0.40, 3.60)] cm, [1.50 (0.70, 3.40)] cm, [2.00 (0.55, 3.90)] cm and [1.30 (0.60, 3.15)] cm. Pain occurred mainly in the shoulder and possibly involved upper limb area (65.1%), and possibly in the surgical site, back and axillary area (34.9%), with a mean score of 5.00 (2.00, 6.00). The limitation of shoulder ROM was manifested in flexion difference of (39.50 ± 23.12)°, extension difference of (13.31 ± 10.16)°, abduction difference of (49.56 ± 27.84)°, horizontal internal rotation difference of [10.00 (0, 39.75)]°, horizontal external rotation difference of [30.00 (2.50, 41.50)]°. The grip strength of the affected side also decreased by [3.50 (1.15, 8.00)]. The main paresthesia was numbness (47.4%), foreign body sensation and pulling sensation around the operative site and axilla (52.6%) (Table 2).

**Table 2:** Degree and extent of lymphedema, pain, limited range of motion and muscle strength, and paresthesia.

Measurements	*N* (%)/mean ± SD/M (P25,P75)
The arm circumference measurement	
0 cm (wrist)	0.80 (0.30, 1.35)
10 cm	1.90 (0.40, 3.60)
20 cm	1.50 (0.70, 3.40)
30 cm	2.00 (0.55, 3.90)
40 cm	1.30 (0.60, 3.15)
Pain	
Position—shoulder and upper limbs	28, 65.1%
Position—chest and back and around axilla	15, 34.9%
NRSs-goal	5.00 (2.00, 6.00)
Shoulder ROM restriction	
Flexion	39.50 ± 23.12
Extension	13.31 ± 10.16
Abduction	49.56 ± 27.84
Horizontal position-internal rotation	10.00 (0, 39.75)
Horizontal position-external rotation	30.00 (2.50, 41.50)
Grip strength restriction	3.50 (1.15, 8.00)
Paresthesia	
Numb	9 (47.4)
Chest and armpit foreign body sensation and pulling sensation	10 (52.6)

*NRSs* numerical rating scales.

### Multiple linear regression of influence on the severity of lymphedema

**Figure 2:**
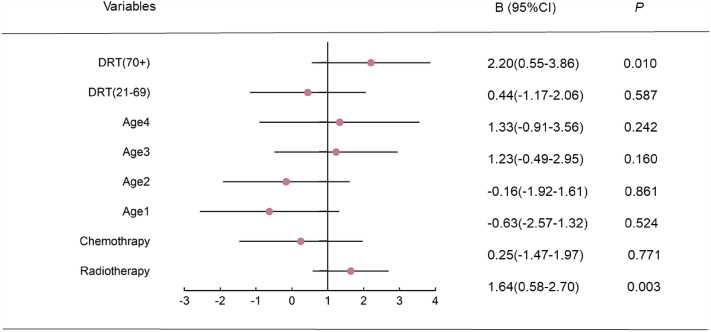
Multiple linear regression of influence on the severity of lymphedema. *DRT* delayed rehabilitation time (month); age 1: 41–50 years’ old; age 2: 51–60 years’ old; age 3: 61–70 years’ old; age 4: 70 + years’ old. R^2^ = 0.22, adjusted R^2^ = 0.17, F = 4.10, *p* < 0.001.

The multifactor linear regression equation was established by including chemotherapy (CT), radiotherapy, age and delayed rehabilitation time. Radiotherapy (RT) significantly increased the degree of edema (B = 1.64, t = 3.06, *p* = 0.003). Compared with patients with a recovery delay of 1 to 20 months, there was no significant difference in the degree of lymphedema in patients with a recovery delay of 21–69 months (B = 0.44, t = 0.54, *p* = 0.587), while there was a significant increase in lymphedema in patients with a recovery delay of more than 70 months (B = 2.20, t = 2.65,* p* = 0.010) (Fig. 2).

### Logistic multifactor regression analysis for the occurrence of lymphedema, pain, shoulder ROM restriction, grip strength restriction and paresthesia

Cancer type (CT) and RT were included to establish a multivariate logistic regression model (MLRM) for the occurrence of lymphedema, with significant contributions from carcinoma in situ (B = -1.8, *p* = 0.003) and RT (B = 1.3, *p* = 0.001). The surgery type (ST) and the occurrence of AWS and BPI were included in the MLRM to establish the occurrence of pain, of which the significant contributions were breast conserving surgery (B = − 2.1, *p* = 0.002) and the occurrence of AWS (B = 3.1, *p* = 0.006). ST and the occurrence of AWS, BPI, pain and edema were included to establish a MLRM of the occurrence of shoulder ROM restriction. The significant contribution was the occurrence of BPI (B = 3.1, *p* < 0.001) and the occurrence of pain (B = 1.9, *p* = 0.002). The occurrence of BPI and pain were included in the MLRM to establish the occurrence of GSR. The significant contribution was the occurrence of BPI (B = 3.6, p < 0.001). The occurrence of BPI and pain were included in the MLRM to establish the occurrence of paresthesia, and the significant contribution was the occurrence of pain (B = 2.1, *p* = 0.001). (Table 3).

**Table 3:** Logistic multifactor regression analysis for the occurrence of lymphedema, pain, shoulder ROM restriction, grip strength restriction and paresthesia.

	Lymphedema AOR (95% CI)	Pain AOR (95% CI)	Shoulder ROM restriction AOR (95% CI)	Grip strength restriction AOR (95% CI)	Paresthesia AOR (95% CI)
Cancer type					
Carcinoma in situ	0.16 (0.05, 0.54)*				
Others	0.20 (0.03, 1.13)				
Radiotherapy (yes)	3.59 (1.64, 7.86)*				
Surgery type					
Modified radical mastectomy		0.39 (0.14–1.11)	0.66 (0.18–2.47)		
Breast conserving surgery		0.12 (0.03–0.45)*	0.52 (0.10–2.92)		
Axillary web syndrome (yes)		0.05 (0.01–0.41)*	1.06 (0.18–6.45)		
Brachial plexus injury (yes)		0.38 (0.11–1.30)	0.04 (0.01–0.21)**	36.38 (7.42, 178.32)**	0.51 (0.13–2.05)
Pain (yes)			6.57 (2.02–21.41)*	0.65 (0.24–1.81)	7.97 (2.35–27.09)*
Lymphedema (yes)			0.49 (0.16–1.46)		

P* < 0.05, p** < 0.001.

## Discussion

It was possible that the prevalence of dysfunction found in the study was underestimated, and there may be a major reason that we do not have access to data from other regions. As far as is currently known, first, some regional medical centers are unable to provide cancer rehabilitation, and they do not have such a department, and do not have the relevant knowledge. Second, clinicians have a weak awareness of referral. They do not recognize the need to assess and treat these sequelae, and do not inform patients of possible complications beyond recurrence after surgery and other treatments^32^. In addition, they are largely unaware of the referral if the patient develops sequelae during follow-up. From the patients, the patients can’t independently identify these long-term chronic complications without prompting from the doctor, for example, they will regard the traumatic lymphedema as a short-term general swelling, believing that "it will get better on its own after a period of time" and thus missing the optimal time for treatment. At present, the common state of breast cancer-related rehabilitation in China is that treatment is likely to be delayed after the occurrence of symptoms, not even to mention how to prevent.

It is important to note that pain and paresthesia are more likely to be ignored than obvious dysfunction such as lymphedema and limited range of motion. Patients usually subjectively ignore this uncomfortable feeling, so they often tell clinicians "we don't have this feeling" in order to show that they are not exceptional. It may also be that after surgical treatment, the patients weren’t informed of the sequelae that may occur in the following period of time, so didn’t treat these problems as symptoms that must be treated. This situation not only happens in China, there are studies that have concluded the common barriers to cancer rehabilitation, such as knowledge barriers including education concerning cancer rehabilitation, access barriers including time, money and transportation and adherence including fear of injury^28^.

In this study, lymphedema was most severe at 10 and 30 cm from the wrist. There were two main "lines" of pain that patients complain about most. One was the surgical site and the affected shoulder extending to the arm, and the other was the affected axilla to the twelfth rib. The limited movement of shoulder joint was mainly manifested in forward flexion and abduction. There was no significant decline in grip strength, except for severe nerve damage from chemotherapy or radiation. The main paresthesia was numbness and pulling. Previous studies had suggested that this series of symptoms can be classified as chemotherapy-induced peripheral neuropathy (CIPN), including numbness, foreign body sensation, tingling, weakness and pain^33^. Until now, it has been difficult to quantify the assessment, most of which was recorded by patients’ self-report, which may lead to an estimate of the occurrence and severity of the symptom.

A large number of previous studies had found that age and BMI were risk factors for lymphedema, but they did not play a significant role in this study. This may be due to the wide age range of the cases we collected and the uniform distribution of the four body index levels. We also found that the type of cancer and the radiotherapy were major risk factors for lymphedema. This also requires further discussion on the classification of cancer types in the following studies. Risk factors affecting the severity of lymphedema were analyzed. Once lymphedema has occurred, older patients may develop higher grades of lymphedema. Treatment with chemotherapy and radiation also increases the degree of lymphedema. In addition to these normally studied factors, the concept of delayed recovery time was added and found to aggravate lymphedema, providing evidence that prevention and early recovery can reduce the occurrence of sequelae.

Although there was no statistically significant association between musculoskeletal symptoms and endocrine therapy in this study, this may be due to the small sample size, we still can’t ignore the possible musculoskeletal sequelae of long-term endocrine therapy. Aromatase inhibitors (AI) are commonly used to treat hormone-receptor-positive breast cancer in postmenopausal women, improving disease-free survival and reducing the incidence of contralateral breast cancer. AI can reduce the synthesis of estrogen, and the decrease of estrogen will lead to the decrease of free calcium concentration, which leads to bone loss and osteoporosis, and also leads to abnormal sensations such as numbness and pain in joints, muscles and bones^34^. Another reason that may have led us to underestimate the effect of endocrine therapy is that we have ignored the time dependence of endocrine therapy. Patients with estrogen-receptor-positive breast cancer who have longer survival are likely to receive long-term endocrine therapy, it will be necessary to incorporate the duration of endocrine therapy into future studies.

We also found that many patients complained about their inability to do normal household chores or take care of their children or grandchild, but rarely brought up problems related to recreational activities, which was different from what had been reported in some European countries. In these studies, the patients would mention that the affected arm can't play golf or tennis/volleyball because it doesn't move well or can't be stretched too much as a precaution^35^. To some extent, this reflects the characteristics of Chinese social life. People, especially some middle-aged and elderly people, are used to focusing on making money and raising children, and they ignore their needs for social entertainment activities, which virtually lowers their requirements for the Qol. This may also be a barrier to breast cancer rehabilitation.

## Conclusion

This study investigated the occurrence and severity of long-term sequelae of breast cancer patients treated in Shanghai, China, and analyzed the relevant risk factors, which were aimed to urge rehabilitation medicine to integrate with other breast cancer treatments and form a sound diagnosis and treatment model. Combined with China's social environment, we need to accurately investigate the actual needs of patients and medical conditions in different regions, so as to clarify the relative treatment mode and points.

## Data Availability

All data generated or analyzed during this study are included in this manuscript.

## References

[1] Sung, H.*, et al.* Global Cancer Statistics 2020: GLOBOCAN Estimates of Incidence and Mortality Worldwide for 36 Cancers in 185 Countries. *CA Cancer J. Clin.* **71**, 209–249 (2021). 10.3322/caac.21660 33538338

[2] Feng, R. M., Zong, Y. N., Cao, S. M. & Xu, R. H. Current cancer situation in China: good or bad news from the 2018 Global Cancer Statistics? *Cancer Commun. (Lond)* **39**, 22 (2019). 10.1186/s40880-019-0368-6 PMC6487510 31030667

[3] DeSantis CE (2016). Breast cancer statistics, 2015: Convergence of incidence rates between black and white women. CA Cancer J. Clin..

[4] Brookham RL, Cudlip AC, Dickerson CR (2018). Quantification of upper limb electromyographic measures and dysfunction of breast cancer survivors during performance of functional dynamic tasks. Clin. Biomech..

[5] Halsted WSI (1907). The results of radical operations for the cure of carcinoma of the breast. Ann. Surg..

[6] Nikkanen TA, Vanharanta H, Helenius-Reunanen H (1978). Swelling of the upper extremity, function and muscle strength of shoulder joint following mastectomy combined with radiotherapy. Ann. Clin. Res..

[7] Patey DH, Dyson WH (1948). The prognosis of carcinoma of the breast in relation to the type of operation performed. Br. J. Cancer.

[8] Madden JL (1965). Modified radical mastectomy. Surg. Gynecol. Obstet..

[9] Shamley D, Srinaganathan R, Oskrochi R, Lascurain-Aguirrebena I, Sugden E (2009). Three-dimensional scapulothoracic motion following treatment for breast cancer. Breast Cancer Res. Treat..

[10] Shamley DR (2007). Changes in shoulder muscle size and activity following treatment for breast cancer. Breast Cancer Res. Treat.

[11] Crosbie J (2010). Effects of mastectomy on shoulder and spinal kinematics during bilateral upper-limb movement. Phys. Ther..

[12] Borstad JD, Szucs KA (2012). Three-dimensional scapula kinematics and shoulder function examined before and after surgical treatment for breast cancer. Hum. Movem. Sci..

[13] Ludewig PM, Reynolds JF (2009). The association of scapular kinematics and glenohumeral joint pathologies. J. Orthopaed. Sports Phys. Ther..

[14] Ebaugh D, Spinelli B, Schmitz KH (2011). Shoulder impairments and their association with symptomatic rotator cuff disease in breast cancer survivors. Med. Hypotheses.

[15] Miaskowski C (2014). Identification of patient subgroups and risk factors for persistent arm/shoulder pain following breast cancer surgery. Eur. J. Oncol. Nurs..

[16] Vidt ME (2012). Characterizing upper limb muscle volume and strength in older adults: A comparison with young adults. J. Biomech..

[17] Sagen, A., Kaaresen, R., Sandvik, L., Thune, I. & Risberg, M.A. Upper limb physical function and adverse effects after breast cancer surgery: A prospective 2.5-year follow-up study and preoperative measures. *Arch. Phys. Med. Rehabil.* **95**, 875–881 (2014). 10.1016/j.apmr.2013.12.015 24389401

[18] Wisotzky E, Hanrahan N, Lione TP, Maltser S (2017). Deconstructing postmastectomy syndrome: Implications for physiatric management. Phys. Med. Rehabil. Clin. N. Am..

[19] Hamood R, Hamood H, Merhasin I, Keinan-Boker L (2018). Chronic pain and other symptoms among breast cancer survivors: Prevalence, predictors, and effects on quality of life. Breast Cancer Res. Treat.

[20] Waltho D, Rockwell G (2016). Post–breast surgery pain syndrome: establishing a consensus for the definition of post-mastectomy pain syndrome to provide a standardized clinical and research approach—a review of the literature and discussion. Can. J. Surg..

[21] Terao Y, Taniguchi K, Fujii M, Moriyama S (2017). Postmastectomy radiation therapy and breast reconstruction with autologous tissue. Breast Cancer.

[22] Johansen S, Fossa K, Nesvold IL, Malinen E, Fossa SD (2014). Arm and shoulder morbidity following surgery and radiotherapy for breast cancer. Acta Oncol..

[23] Levy EW (2012). Predictors of functional shoulder recovery at 1 and 12 months after breast cancer surgery. Breast Cancer Res. Treat.

[24] Wernicke AG (2011). A 10-year follow-up of treatment outcomes in patients with early stage breast cancer and clinically negative axillary nodes treated with tangential breast irradiation following sentinel lymph node dissection or axillary clearance. Breast Cancer Res. Treat.

[25] Monleon S (2016). Shoulder strength changes one year after axillary lymph node dissection or sentinel lymph node biopsy in patients with breast cancer. Arch. Phys. Med. Rehabil..

[26] Yang JD (2015). Physical and functional ability recovery patterns and quality of life after immediate autologous latissimus dorsi breast reconstruction: A 1-year prospective observational study. Plast. Reconstr. Surg..

[27] Boquiren VM (2016). A longitudinal analysis of chronic arm morbidity following breast cancer surgery. Breast Cancer Res. Treat..

[28] Stubblefield MD (2017). The underutilization of rehabilitation to treat physical impairments in breast cancer survivors. PM R.

[29] Clerke AM, Clerke JP, Adams RD (2005). Effects of hand shape on maximal isometric grip strength and its reliability in teenagers. J. Hand. Ther..

[30] Williamson A, Hoggart B (2005). Pain: A review of three commonly used pain rating scales. J. Clin. Nurs..

[31] Thong ISK, Jensen MP, Miro J, Tan G (2018). The validity of pain intensity measures: What do the NRS, VAS, VRS, and FPS-R measure?. Scand. J. Pain.

[32] Rafn BS, Midtgaard J, Camp PG, Campbell KL (2020). Shared concern with current breast cancer rehabilitation services: a focus group study of survivors' and professionals' experiences and preferences for rehabilitation care delivery. BMJ Open.

[33] Brown TJ, Sedhom R, Gupta A (2019). Chemotherapy-induced peripheral neuropathy. JAMA Oncol..

[34] Winters L, Habin K, Gallagher J (2007). Aromatase inhibitors and musculoskeletal pain in patients with breast cancer. Clin. J. Oncol. Nurs..

[35] Lattanzi JB (2010). Recommendations for physical and occupational therapy practice from the perspective of clients undergoing therapy for breast cancer-related impairments. J. Allied Health.

